# The Interplay among Polyamines and Nitrogen in Plant Stress Responses

**DOI:** 10.3390/plants8090315

**Published:** 2019-08-30

**Authors:** Konstantinos Paschalidis, Georgios Tsaniklidis, Bao-Quan Wang, Costas Delis, Emmanouil Trantas, Konstantinos Loulakakis, Muhammad Makky, Panagiotis F. Sarris, Filippos Ververidis, Ji-Hong Liu

**Affiliations:** 1 Department of Agriculture, School of Agricultural Sciences, Hellenic Mediterranean University, Estavromenos, GR-71500 Heraklion, Greece; 2 National Agricultural Research Foundation (NAGREF), GR-71103 Heraklion, Greece; 3 School of Horticulture and Landscape Architecture, Henan Province Engineering Research Center of Horticultural Plant Resource Utilization and Germplasm Enhancement, Henan Institute of Science and Technology, Xinxiang 453003, China; 4 Department of Agriculture, University of the Peloponnese, GR-24100 Kalamata, Greece; 5 Department of Agricultural Engineering, Universitas Andalas, Padang 25163, Indonesia; 6 Biosciences, University of Exeter, Geoffrey Pope Building, Exeter EX4 4QD, UK; 7 Institute of Molecular Biology & Biotechnology, GR-70013 Heraklion, Greece; 8 Department of Biology, University of Crete, GR-70013 Heraklion, Greece; 9 Key Laboratory of Horticultural Plant Biology, College of Horticulture and Forestry Sciences, Huazhong Agricultural University, Wuhan 430070, China

**Keywords:** polyamines, nitrogen metabolism, abiotic and biotic stress, hydrogen peroxide, antioxidant machinery

## Abstract

The interplay between polyamines (PAs) and nitrogen (N) is emerging as a key factor in plant response to abiotic and biotic stresses. The PA/N interplay in plants connects N metabolism, carbon (C) fixation, and secondary metabolism pathways. Glutamate, a pivotal N-containing molecule, is responsible for the biosynthesis of proline (Pro), arginine (Arg) and ornithine (Orn) and constitutes a main common pathway for PAs and C/N assimilation/incorporation implicated in various stresses. PAs and their derivatives are important signaling molecules, as they act largely by protecting and preserving the function/structure of cells in response to stresses. Use of different research approaches, such as generation of transgenic plants with modified intracellular N and PA homeostasis, has helped to elucidate a plethora of PA roles, underpinning their function as a major player in plant stress responses. In this context, a range of transgenic plants over-or under-expressing N/PA metabolic genes has been developed in an effort to decipher their implication in stress signaling. The current review describes how N and PAs regulate plant growth and facilitate crop acclimatization to adverse environments in an attempt to further elucidate the N-PAs interplay against abiotic and biotic stresses, as well as the mechanisms controlling N-PA genes/enzymes and metabolites.

## 1. Introduction

Nitrogen metabolites, polyamines (PAs), and several important plant phytohormones, such as ethylene, jasmonates, abscisic acid, salicylic acid, have shown to act as crucial growth regulators that can cross talk with each other in stress signaling processes [1,2,3,4,5,6,7,8,9,10,11,12,13,14,15,16,17,18,19,20,21,22]. PA biosynthesis/degradation and their homeostasis undergo extensive alterations in response to various stress conditions, such as cell wall degradation [23] oxidative and developmental stress [2,24,25,26,27,28,29,30,31], phytopathogenic bacteria/fungi/viruses [30], water deficiency [31,32,33], ammonia toxicity and nutrient availability [34], salinity [2,35,36,37] and heat [38,39].

Plants absorb nitrogen (N) mostly as nitrate or ammonia ions. The nitrate molecules are enzymically converted to ammonia, which is assimilated in plants for amino acid synthesis. Ammonia assimilation is mainly catalyzed by the glutamine synthetase (GS)/glutamate synthase (GOGAT) cycle [40]. Ammonia detoxification, on the other hand, is catalyzed mainly by glutamate dehydrogenase (GDH); however, under stress conditions GDH partly contributes to ammonia assimilation [34,41]. Furthermore, accumulating evidence shows that stress induces PA export and subsequent oxidations in the apoplast that play a role in production of H_2_O_2_ [22,30,42]. The apoplastic polyamine oxidase (PAO) in cooperation with the NADPH-oxidase creates a feedforward reactive oxygen species (ROS) magnification loop, affecting the oxidative status and climaxes in programmed cell death (PCD) performance. This loop may be a crucial point in many reactions governing salinity stress resistance, with possible functions spreading outside stress resistance [36]. In tobacco transgenic plants overexpressing *ZmPAO*, we detected greater apoplastic/cytoplasmic contents of H_2_O_2_ and superoxide, accompanied by increase in antioxidant genes; however, these antioxidants cannot efficiently scavenge ROS [27,28]. On the other hand, repression of *ZmPAO* in young tobacco seedlings enables them to resist short-term salinity, which can be attributed either to higher PA content or to lower ROS contents, because of the perturbed PA apoplastic oxidation [27,28].

The links between PAs and growth-regulatory pathways at molecular, biochemical and physiological levels, suggest that altering the expression of specific PA-response factors could provide a new strategy for targeted PA-response engineering. This review elucidates the concerted roles of N and PAs against plant abiotic and biotic stressors, as well as their interplay mechanisms, as far as the related genes/enzymes and metabolites are concerned, in order to help plants adapt to unfavorable environmental conditions.

## 2. Major Genes Involved in Abiotic and Biotic Stress Responses

It has been documented that drought and salinity, two main abiotic stress factors, disturb at least 20% of the arable land and nearly 40% of the irrigated land in the world [43,44]. These factors severely limit crop yields and result in the loss of more than US$100 billion per annum to the agricultural sector [45]. Drought represents a reduced soil water capacity, causing a decrease in root water uptake, while salinity leads to enhanced salt ion levels in the soil. Because of both lower water potential in the soil and higher ion levels, higher osmosis in plant cells/tissues might lead to elevated levels of osmolytes in order to achieve osmotic balancing. Furthermore, the higher concentration of ions inside the plant may induce an ionic chain reaction, which increases with the duration of the stress, leading to endocellular permeability of toxic ions. In order to combat with these harsh conditions, the plant can respond by either excluding ions or compartmentalizing them in vacuoles. In this regard, the osmotic phenomenon occurs very quickly and is found in all stress conditions, whereas the ionic phenomenon is quite long, progresses with time, and only under salinity [46]. In comparison to abiotic stress, several biotic stress factors, such as viruses, bacteria, fungi, nematodes, insects, and weeds, cause a direct deprivation of plant nutritional agents, leading to decreased strength in host plants. In agricultural terms, both biotic and abiotic stresses cause dramatical pre- and postharvest damages [47].

Plants adopt specific morphological and cellular alterations by sensing stress signals in order to adapt to environmental conditions. However, very few presumed sensors have been recognized. This is mainly due to the fact that functional genes encoding sensor proteins may exist in a redundant way, so alteration of one gene does not cause substantial phenotype alterations under stress. Alternatively, a sensor protein may be essential to plants and loss-of-function mutants are lethal to plants, prohibiting further analysis [43].

Numerous sets of genes/products are linked to abiotic/biotic stress responses at transcriptional and translational level. Genes leading to effective plant adaptation/tolerance may be categorized into four major groups: (i) Genes coding for enzymes of osmolyte biosynthesis, such as proline, mannitol, glycine, betaine, and trehalose; (ii) genes coding for antioxidant enzymes, such as superoxide dismutase (SOD), peroxidase (POD), and catalase (CAT); (iii) genes coding for stress-induced proteins, such as antifreeze proteins, chaperons, and heat shock proteins; and (iv) genes coding for regulatory proteins, such as protein kinases and transcription factors [44]. So far, a large number of functional and regulatory genes implicated in abiotic and biotic stress responses have been identified in a diversity of plants. In particular, we characterized some functional or regulatory genes controlling metabolism of N, PAs, ethylene, and abscisic acid that are involved in various biological processes [22,33,48,49,50,51,52,53,54,55,56,57,58,59,60,61,62,63,64,65,66,67,68,69,70,71,72].

## 3. Stress-Related Nitrogen Flow and Polyamines

**Figure 1:**
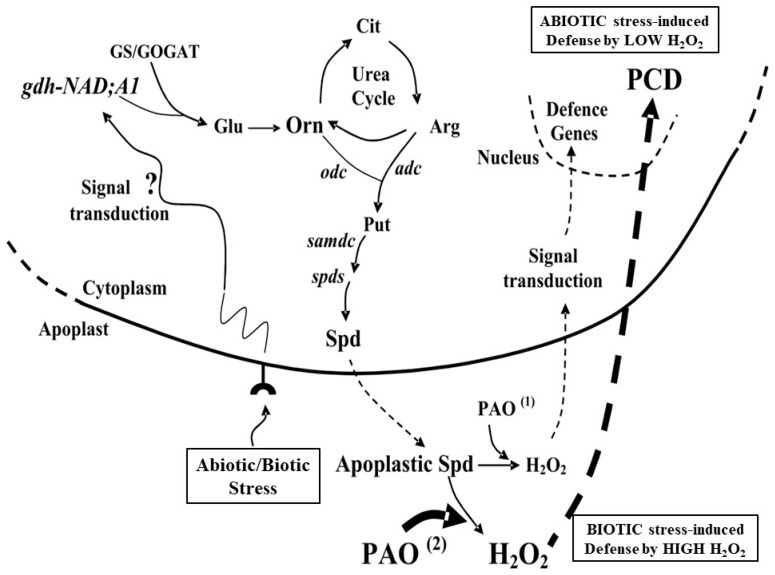
Proposed model for the nitrogen–polyamine (N-PA) interplay in plant abiotic/biotic stress signaling and defense. Abiotic or biotic stress induces proteases activity and increases in ammonium ions inside the cell. The reactive oxygen species (ROS) stress signal triggers the induction of the gene encoding the α-subunit of glutamate dehydrogenase (gdh-NAD; A1), which contributes, together with the GS/GOGAT cycle, to ammonia assimilation [34,41]. Stress-induced glutamate (Glu) production by GDH is diverted to Pro biosynthesis. Stressful conditions cause a further increase in endocellular PAs that are excreted and apoplastically oxidized by polyamine oxidase (PAOs), thus, producing H_2_O_2_ and numerous N composites. Depending on the level of H_2_O_2_ produced under abiotic stresses, programmed cell death (PCD; high H_2_O_2_ levels above a certain threshold) or H_2_O_2_ scavenging (low levels below a certain threshold) is activated [22,27,28,33]. However, the biotic stress-induced H_2_O_2_ causes a reverse pattern, as high H_2_O_2_ levels form an apoplastic “barrier” protecting the plant from fungi and bacteria [30]. Ascorbate peroxidase (APX) and other antioxidant genes are also involved during the protection response. Moreover, PAs are peroxisomally back-converted to generate H_2_O_2_ and N compounds that could activate Ca^2+^ permeable channels [2,22,28,32,73,97]. PAO ^(1)^: Decrease of PAO activity results in increased Spd and Spm contents and low levels of H_2_O_2_, leading to expression of defense genes and plant tolerance to abiotic stress, but susceptibility to biotic stress (fungi and bacteria); PAO ^(2)^: Increase of PAO activity results in lower Spd and Spm contents and high levels of H_2_O_2_, leading to abiotic stress-induced PCD accompanied by plant abiotic stress susceptibility, but tolerance to biotic stress due to high levels of H_2_O_2_, which form a “barrier” to fungi and bacteria. The other abbreviations are found in the text.

Nitrogen is one of the main essential nutrient elements in plants. The N molecules inside plant cells are derived from soil inorganic N uptake, usually in the form of nitrate and ammonium ions, and from ammonia assimilation, N transport throughout the plant, and N remobilization (Figure 1) [34,73,74,75,76,77,78].

Nitrogen levels influence plant productivity and quality, due to association with various growth substances that are involved in plant stress responses [31,34,55,79,80]. PAs often increase in plants as a result of N application. They usually result from N-induced higher concentrations of their precursor amino acids, such as Orn and Arg, which are converted to putrescine (Put) [20,31,81]. The interplay among PAs and N is evolving as a major participant in plant stress reactions. The first synthesized amino acid is commonly glutamate (Glu), which participates in N recycling/remobilization into other nitrogenous molecules, ensuring N homeostasis in plants (Figure 1). The Glu, as a central N molecule, leads to biosynthesis of proline (Pro), Orn, Arg, and PAs, which constitute a crucial cooperating pathway for carbon (C) and nitrogen (N) assimilation [78]. Pro, Arg, and Put concentrations in plants are further known as some of the important indicators for both biotic and abiotic stress response [31]. *S*-adenosylmethionine (SAM), which is formed by methionine and involved in ethylene biosynthesis, and Orn, an amino acid involved in the urea cycle, are two important precursor molecules in PA synthesis (Figure 1). Put, Spd, Spm, and thermospermine, in turn, are important products of the organic N, as they are found at relatively high endogenous levels. PAs and their C scaffold are involved in several biochemical pathways. PA catabolism has a crucial role in N/C assimilation/remobilization, as it recycles C and N and produces H_2_O_2_ by PAO [22,26,39,42,73,78]. However, PA accumulation inside plant cells is the consequence of biosynthesis/catabolism, inter-conversions, and conjugation.

Carbon and N are crucial for developmental and stress responses in plants, in terms of life cycle accomplishment and crop production. Therefore, an appropriate N/C balancing is extremely critical for a variety of physiological and biological processes, including stress response. However, the N/C signaling machineries remain fundamentally undiscovered. The N/C cooperative genome-wide function has revealed that the majority of genes in *Arabidopsis* are over- or under-controlled by C and/or N input [78]. Furthermore, PA remobilization is related with the nitrate transport in parenchymal shoot tissues [82]. PA catabolism, producing H_2_O_2_ and GABA in the cell wall, is also tightly involved in preserving the N/C homeostasis and balance inside plant tissues [83].

It is widely accepted that the PA/N interplay in plants is of major interest, because it connects N metabolism, C fixation, and secondary metabolism pathways. Stress conditions, such as salinity and drought, increase the activity of proteases causing augmentation of ammonium ions inside cells (Figure 1). Ammonia is converted into glutamine and Glu by GS/GOGAT, respectively. Glu gives Orn that requires higher PA biosynthesis in response to various stressful conditions [41].

Orn is a key amino acid participating in cooperating pathways with major amino acids (Figure 1). Transgenic mouse ornithine decarboxylase (mODC) plants with depleted Orn exhibited higher Glu/Orn conversion into Put, leading to N shortage in the cell and to decreased protein synthesis. The same plants also transformed more Glu into Orn, which was partially compensated for enhanced Glu synthesis from integrated N and C [78]. Therefore, overall N assimilation/partitioning in plants is largely dependent on C availability/reallocation and vice versa. Under threatening abiotic and biotic stress conditions, the plants respond by remobilizing N and C into signaling molecules, such as PAs, Pro, GABA, glycine betaine, and β-Ala [78], as they have stress-protective key roles and partly alleviate ammonia cell toxicity. A Glu-Pro-Arg-PA-GABA coordinated path is therefore of major importance to accomplish an equilibrium among assimilated/partitioned N/C inside plant cells [34,78]. Proteomics and transcriptomics studies on PA-stress interaction and classification of major proteins implicated in important plant developmental/stress responses may provide new insights into the molecular mechanisms underlying these processes [84,85]. Moreover, by RNA-RNA in situ hybridization (ISH) methodologies, we have further elucidated the functions of N/PA genes in crop plants. Use of ISH has helped to identify the localization of PA anabolic and catabolic gene transcripts in tissues, such as the locular parenchyma and the vascular bundles, supporting the viewpoint that Put biosynthetic and catabolic genes are mostly expressed in fast growing tissues and that PAs are strongly implicated in fruit ripening [20].

## 4. N/PA Biotechnological Approaches for Enhanced Tolerance to Abiotic and Biotic Stress

Plants usually circumvent stress conditions by stimulating appropriate responses that lead to altered metabolism and growth. Tolerance to abiotic stress conditions might be achieved via genetic engineering through modifying the endogenous concentrations of osmoprotectants, by increasing ROS scavenging capacity or by robustly excluding ions with efficient transporter/symporter systems [86]. Taking into account that dissecting the function of stress-related genes would assist in elucidating the potential biochemical and molecular machineries for stress adaptation, enormous efforts and approaches have been expended to unravel the genes/proteins/metabolites associated with a plethora of cellular processes that regulate the complicated character of abiotic and biotic stress resistance [44].

As PAs have pleiotropic roles, their homeostasis control is complex. Genetic transformation of N assimilation/detoxification genes and PA biosynthetic genes coding for GDH, arginine decarboxylase (ADC), ODC, SAM decarboxylase (SAMDC), or Spd synthase (SPDS), significantly enhances abiotic stress resistance in numerous plant species [4,5,34,36,37,38,39,41,63,72,87,88,89,90,91,92,93,94,95,96].

The GS/GOGAT pathway is the main ammonia assimilation cycle in plants. However, under stress conditions, photorespiratory ammonia may hyperaccumulate due to decreased activity of GOGAT or GS. In this regard, we have discovered alternative metabolic ways like GDH that are activated in order to decrease ammonia buildup in the cells (Figure 1) [34]. Transgenic tobacco plants overexpressing the plant *GDH* gene encoding for the a-subunit polypeptide of GDH (gdh-NAD;A1) also exhibit higher ammonium assimilation activity [41]. Furthermore, transgenic rice overexpressing the *GDH* gene from *Eurotium cheralieri* (a lower organism that has stronger ammonium affinity compared to higher plants) showed higher N assimilation efficacy and yield, especially under low N conditions [87].

Under abiotic stress environment, PAs are apoplastically delivered and oxidized by PAO (Figure 1), generating several intermediates. We revealed two different outputs based on the level of the PA oxidized products. On the one hand, low apoplastic PAO generates less amount of H_2_O_2_, which in turn initiates a ROS protective pathway that triggers tolerance reactions. On the other hand, high apoplastic PAO could produce a large level of H_2_O_2_, thus triggering plant cell death (PCD) [22,28,42].

The above scenario illuminates mainly the intercellular PAs’ role. In another study, transgenic tobacco plants with down-regulated SAMDC underwent abiotic stress-induced PCD, and displayed lower endocellular levels of soluble Spd and Spm. However, we found that PA contents and apoplastic oxidation in the transgenic plants were unpredictably comparable to those of the wild type [28]. The down-regulated SAMDC transgenics, thus, present a balanced PA interplay among developmental and stress responses [32].

However, during biotic stress an opposite scenario is observed. In the *PAO*-overexpressing tobacco plants, we detected a stress-induced up-regulation of the *PAO* gene upon exposure to infection by *Pseudomonas syringae* pv tabaci [30]. The increased expression of the *ADC* gene may promote PA stability. Spm, in turn, is apoplastically excreted and broken down by the enhanced *PAO*, generating excess H_2_O_2_ that helps plants to cope with the pathogen attack [30]. Therefore, transgenic plants with increased *PAO* exhibited pre-induced resistance towards infections, including biotrophic and hemibiotrophic diseases [30]. Our stress defense model may represent a pioneering way for creating transgenic plants resistant to both abiotic and biotic stresses.

PA catabolism in plants plays a key role in the antioxidant machinery under stress conditions. Overexpression of an apoplastic *PAO* in tobacco plants led to higher expression of antioxidant machinery, including SOD and CAT [27]. However, the induced machinery did not conclude in stress defense, as it represented an effort to neutralize the PAO-produced H_2_O_2_. Thus, we suggested that continuous PA oxidation may lead to a continuous stress condition. The same genetically modified *Nicotiana tabacum* plants with altered PA/H_2_O_2_ levels due to over/underexpression of the *ZmPAO* gene were examined under heat stress. When the *ZmPAO* gene was repressed in transgenic plants, they exhibited better thermotolerance, higher biomass growth, and higher enzymatic and non-enzymatic antioxidant levels. In contrast, the *ZmPAO*-overexpressing plants showed a compromised thermotolerance [38]. Moreover, the *ZmPAO*-underexpressing plants possessed higher Ca^2+^ levels with salinity, associated with lower chlorophyll levels, leaf area and biomass, and a taller phenotype, than the wild type. The *ZmPAO*-overexpressing plants, on the contrary, had a higher number of leaves with slightly greater size and higher antioxidant genes/enzyme levels than the underexpressing ones [37]. Therefore, different phenotypes are found in *PAO*-overexpressing and underexpressing plants under abiotic/biotic stress conditions, revealing a multifaceted character of the apoplastic PAO. In this regard, PAO exerts an important role in rendering plants to survive under both abiotic and biotic stress conditions. It is further proposed that the PAO/NADPH oxidase loop is a focal point in the control of several defense processes in plants, including stress tolerance (Figure 1) [22,24,25,35,36].

## 5. Conclusion and Perspectives

Metabolic engineering has great potential to enhance abiotic and biotic stress tolerance. Many compounds play dynamic roles in incorporating stress signals, regulating stress response through modifying gene expressions and controlling numerous transporters and biochemical pathways in plants. Modification of a single step in the N/PA cycle (e.g., elevated Glu synthesis via transgenic *GDH* and/or increase of a specific PA via overexpressing of the respective PA biosynthetic gene) might cause a substantial redistribution of the metabolome in the cell. In this regard, for model plants (e.g., *Arabidopsis*) [35,98,99] or plants of industrial use (e.g., grapevine, tobacco, tomato, citrus, etc.) [22,27,28,29,30,33,34,37,38,39,41], metabolic engineering to alter N/PA metabolism and the accompanying N/C accumulation might provide a suitable means for elucidating the physiological mechanisms underlying the increase in crop yield and quality under stress conditions. Extensive knowledge of the N-PA crosstalk by means of engineering technologies may further open new avenues or suggest alternative possibilities for improving the quality of agricultural food products with additional paybacks, such as nutraceuticals and functional components.
